# Analyzing the cytotoxicity and pro‐tumorigenic role of acetyl tributyl citrate in prostate through network toxicology and the immune microenvironment

**DOI:** 10.3389/fimmu.2026.1758050

**Published:** 2026-09-08

**Authors:** Shangyan Xie, Mingsong Qing, Zihong Zhang, Hang Zhang, Yunxiao Zhang, Zhenyu Yang, Zhiwei Chen, Zhuozhi Liang, Garu A

**Affiliations:** 1 Department of Orthopedic Surgery, Foshan Fosun Chancheng Hospital, Foshan, China; 2 Department of Orthopaedic Surgery, The Kweichow Moutai Hospital, Renhuai, China; 3 Department of Orthopaedic Surgery, The Second Affiliated Hospital of Guangzhou Medical University, Guangdong, China

**Keywords:** acetyl tributyl citrate, immunotherapy, machine learning algorithms, prostate cancer, tumor microenvironment

## Abstract

**Background:**

Acetyl tributyl citrate (ATBC) is an alternative plasticizer to Di(2-ethylhexyl) phthalate (DEHP) and bisphenol A (BPA) that may contribute to the development of various cancers. The cytotoxic activity and pro‐tumorigenic role of ATBC in prostate cancer (PCa) progression remains poorly understood, highlighting the need for a systematic evaluation of its molecular toxicity.

**Methods:**

This study identified potential targets of ATBC in PCa by integrating multiple online databases, including GEO, ChEMBL, STITCH, and Swiss Target Prediction. Protein-protein interaction (PPI) analysis was performed on the intersecting targets, followed by Gene Ontology (GO), Kyoto Encyclopedia of Genes and Genomes (KEGG) functional enrichment, and immune microenvironment analyses. Subsequent these crucial targets were identified through the application of machine learning algorithms, specifically the Support Vector Machine-Recursive Feature Elimination (SVM-RFE) and Least Absolute Shrinkage and Selection Operator (LASSO). The expression of these targets was validated using external datasets from The Cancer Genome Atlas (TCGA) and *in vitro* experiments. Molecular docking analysis was used to explore the interactions between the key genes and ATBC. Finally, virtual knockout and pathway enrichment analysis simulations on single-cell RNA-seq data were conducted to investigate the biological functions of these genes.

**Results:**

Through the utilization of multiple online databases, we identified 45 potential targets associated with ATBC exposure-related PCa. Further refinement using STRING and Cytoscape software elucidated the PPI network. GO and KEGG pathway analyses revealed that these targets, associated with ATBC and PCa, were predominantly enriched in several signal pathways. Subsequent analysis employing machine learning algorithms identified three core targets: EZH2, DNMT1, and PRMT5, which influence the immune infiltration of PCa. Additionally, molecular docking analysis and *in vitro* experiments further confirmed these potential therapeutic targets. Virtual knockdown of DNMT1, EZH2, and PRMT5 reveals their essential role in preserving collagen-rich ECM integrity and cell–matrix adhesion to maintain prostatic stromal homeostasis, whose disruption mediates ATBC-induced cytotoxicity and potential pro-tumorigenic effects.

**Conclusion:**

Overall, the findings provide new insights into linking the effects of ATBC to PCa, and they provide a preventive and therapeutic strategy for PCa that exposure to the environments with excessive ATBC levels.

## Introduction

DEHP and BPA are common plasticizers found in various products, including cosmetics, medications, and medical packaging (1). These substances can be detected in human tissues and urine are known to cause oxidative stress, cell apoptosis, and metabolic disruptions *in vitro (*2, 3). In order to overcome this limitation and reduce the associated risks, ATBC is considered a viable substitute. ATBC, recognized as a safe plasticizer, exhibits a remarkably wide and diverse range of applications (4). Currently, it is extensively utilized in adhesives, coating formulations, and medical equipment (5). Despite ATBC is a widely utilized alternative plasticizer, its mobility poses potential exposure risks. This is due to the lack of stable bonding between ATBC and plastic polymers, which can result in its leaching from food packaging, medical devices, and other plastic products. A significant concern pertains to ATBC is found in relatively high concentrations in indoor dust, with levels reaching 13,800 ng/g in China (6). Its environmental impact is becoming more apparent in multiple aquatic systems throughout the world, including Kuwait (0.05 μg/l), Korea (0.096 μg/l), Germany (0.54 μg/l), and UK (154 μg/l) (7–9). Approximately 99% of ATBC is excreted through urine, feces, or exhaled air (10). Some studies have demonstrated that ATBC, at a concentration of 4.02 μg/mL, can induce the upregulation of cytochrome P4503A4 levels and enzyme activity in human intestinal cells (11). Besides, another studies that low-dose ATBC (100 μM) significantly inhibited MC3T3-E1 cell proliferation and migration (12). ATBC inhibits BMSC cells’ ability to differentiate into bone and reduces calcium nodule formation. In rats, six weeks of daily ATBC exposure at 743.4 ng/kg led to osteoporosis, evidenced by cortical bone thinning and trabecular bone loss, as shown by micro-CT scans. Quantitative analysis confirmed significant declines in bone quality and microstructural parameters. These results suggest that prolonged ATBC exposure can cause osteoporosis by hindering bone formation and altering bone structure (13). Furthermore, additional studies have suggested that ATBC may contribute to the progression of bone cancer and oral squamous cell carcinoma by targeting critical signaling pathways and altering the tumor microenvironment (14, 15). These findings highlight the potential adverse effects of ATBC on multiple systems.

PCa represents a significant global health concern, characterized by a multifactorial etiology with well-established risk factors including age, race, and family history (16, 17). A most common complication associated with PCa is bone metastasis, occurring in approximately 80% of individuals with advanced stages of the disease (18). The incidence and progression of PCa are closely linked to the presence of androgens and the activation of the androgen receptor (AR), androgen deprivation therapy is commonly employed as the standard treatment for patients with bone metastases (19, 20). Recent studies have identified ATBC have potential endocrine-disrupting effects. Specifically, ATBC has been found to exhibit both estrogen receptor antagonist activity and androgen receptor agonist characteristics (21, 22). As an alternative plasticizer, ATBC is generally considered safe at typical concentrations. However, there is evidence suggests that ATBC may damage the reproductive system by reducing cell viability in TM3 cells (23). Furthermore, a recent study has identified the presence of microplastics in both para-tumor and tumor tissues of the human prostate (24). Nevertheless, the relationship between PCa progression and microplastics, particularly ATBC, remains unclear. Because of the pivotal role of hormone in PCa, we hypothesize that ATBC may compromise prostate function via influencing androgen levels, potentially contributing to the development and progression of PCa. Consequently, it is imperative to elucidate the intricate toxicological pathways of ATBC in relation to PCa. To this end, we have undertaken a comprehensive network toxicology analysis to explain its potential toxicity and molecular mechanisms.

Network toxicology integrates toxicology and bioinformatics to transform complex toxicity mechanisms into succinct and understandable models, including toxic components, targets, and pathways. Molecular docking (MD), originally developed as a screening tool to aid drug design, is adept at modeling toxic components-targets to predict binding site and binding energies (25). In summary, these methodologies facilitate an efficient and adequate evaluation of toxins and elucidate the potential mechanisms. In this study, in order to elucidate the complex toxicological pathways of ATBC in relation to PCa, we conducted a comprehensive network toxicology analysis and *in vitro* experiments to uncover its potential toxicity and molecular mechanisms. This work improves our understanding of environmental pollutant toxicity and lays the groundwork for strategies to address illnesses related to ATBC exposure.

## Materials and methods

### Cell culture

The human prostate stromal cells WPMY-1 cells (Procell Biotechnology, Wuhan, China) were maintained in DMEM Medium supplemented with 10% FBS and 1% antibiotics at 37°C in a 5% CO_2_ humidified atmosphere. The PC-3 cells (Procell Biotechnology, Wuhan, China) were maintained in Ham’s F-12K Medium supplemented with 10% FBS and 1% antibiotics at 37°C in a 5% CO_2_ humidified atmosphere.

### Selection of PCa-related targets

The GSE38241, GSE27616, GSE6956 dataset was obtained from the Gene Expression Omnibus (GEO, https://www.ncbi.nlm.nih.gov/geo/) database. The GSE38241 dataset included 18 PCa samples and 21 normal prostate samples, and GSE6956 included 20 prostate normal tissues and 69 PCa samples, while GSE27616 had 4 normal prostate and 9 PCa samples (**Supplementary S1**). The R programming language was utilized to identify Differentially expressed genes (DEGs). Initially, data normalization and background correction were analyzed using ‘normalizeBetweenArray’ function within the ‘limma’ package. Following this, differential expression analysis (*padj* < *0.05* & |*logFC*| >*1*) was conducted employing the Limma package. The heatmap and volcano plot were created to display the top 50 DEGs with the most significant variations.

### Collection of ATBC targets

The standard molecular structure and SMILES notation of “acetyl tributyl citrate” were determined by querying the PubChem database (https://pubchem.ncbi.nlm.nih.gov/), we identified potential targets of ATBC from the ChEMBL dataset(https://www.ebi.ac.uk/chembl/) by employing the same keyword, with the search parameters restricted to “Homo sapiens” to ensure biological relevance. The SMILES code for ATBC was subsequently submitted to the STITCH database (https://ngdc.cncb.ac.cn/databasecommons/database/id/208) to explore additional potential targets that might have been overlooked. In addition, potential targets for ATBC were further identified using Swiss Target Prediction (http://www.swisstargetprediction.ch/). We meticulously verified the structural integrity and consistency of the search results, eliminating any duplicates. We then consolidated the data from Swiss Target Prediction, ChEMBL, and STITCH to compile a comprehensive database of verified targets for ATBC.

### PPI network construction

We employed “Venn “ package to identify hub genes from the intersecting ATBC target networks and PCa targets. The 45 overlapping genes were then imported into the STRING (https://cn.string-db.org/) to construct PPI network, with an interaction confidence threshold set at greater than 0.4. The resulting PPI network was imported into Cytoscape (v3.8.2) for detailed topological analysis. Clustering analysis with the Degree plugin in Cytoscape identified highly interconnected functional modules, identifying the top 10 hub targets.

### GO and KEGG enrichment analyses

To investigate the biological mechanisms of ATBC in PCa, we performed a GO function and KEGG pathway enrichment analysis for the intersecting genes utilizing the “GO plot” package in R software (Version: 4.2.3), thereby providing insights into the roles of potential targets. The enrichment GO terms and pathways were ordered according to the gene ratio, and the p value less than 0.05 were considered statistically significant. Finally, the results were visualized using bar plots and bubble charts to enhance clarity.

### Screening and validating of feature genes

The identification of feature genes was conducted using the LASSO and the SVM-RFE machine learning algorithms. Machine learning represents an innovative analytical tool for algorithmic analysis. In this study, the LASSO and SVM-RFE algorithms were utilized for marker screening. LASSO regression was executed using the “glmnet” R package, with 10-fold cross-validation applied to hub genes. Furthermore, the SVM-RFE model was developed using the “e1071” SVM function to ascertain the optimal number of ranked genes. Ultimately, the intersection of results from these machine learning algorithms was validated using the prostate Adenocarcinoma (TCGA-PRAD) dataset. In order to evaluate the differences in expression levels between these two sample types, Wilcoxon rank-sum tests were employed, resulting in the calculation of corresponding p-values. To validate these feature genes, an independent validation analysis was performed using the GSE32269 dataset.

### Analyzes of immune infiltration

The “CIBERSORT” package was employed to evaluate the proportions of various immune cells in PCa samples. The boxplots and barplots were generated by “ggplot2” and “ggpubr” packages. Furthermore, correlation analysis was performed to investigate the relationship among the expression of feature genes and immune cells in PCa. Correlation heat map was constructed to examine the relationship between EZH2, DNMT1, and PRMT5 expression levels and immune cell infiltration levels.

### Single-cell RNA sequencing analysis and virtual knockdown

This research utilized single-cell RNA sequencing data from the GSE143791 dataset in the TISCH (http://tisch.comp-genomics.org/) database, which specializes in tumor microenvironment studies. The GSE143791 contains a total of 41 samples, which were obtained from different tissue components of prostate cancer patients, including solid tumor metastases, involved bone marrow, distant bone marrow, and Benign. The raw data were normalized, and cells with fewer than 500 or more than 4,000 genes per cell were excluded. Dimensionality reduction was performed using PCA, followed by batch correction and cell visualization via UMAP. This study focused on the expression of EZH2, DNMT1, and PRMT5 in various immune cell types in PCa. To infer the functional impact of the hub genes, we performed in silico knock down of the three hub genes using scTenifoldKnk. For each hub gene, we removed it from the network, recalculated the edge weights, and ranked the genes by a composite score (|Z-score| × network distance). Finally, the top 20 perturbed genes were subjected to GO and KEGG enrichment analyses (*p < 0.05*).

### CCK-8 assay

ATBC (Catalog number HY-N0145, MCE) was dissolved in dimethyl sulfoxide (DMSO) to prepare a 1 M stock solution. Working concentrations (0, 2.5, 5, 10, 20, and 40 μM) were prepared by diluting the stock in complete culture medium, with the DMSO volume held constant across all treatment groups. The cell viability was tested using CCK-8. PC-3 and WPMY-1 cells were seeded at a density of 3000 cells per well in 96-well plates and incubated for 24, 48, and 72 hours. The experiment was performed independently five times (n = 5). Following the initial incubation period, the cells were treated with different concentrations of ATBC (0, 2.5, 5, 10, 20, and 40 µM). Subsequently, the culture medium was replaced with 100 µL of fresh medium supplemented with 10 μL of CCK-8 solution per well. The plates were then incubated at 37°C for an additional 2 hours. Absorbance at 450 nm was measured using a Bio-Tek microplate reader (Winooski, VT, USA). Five wells were randomly selected for each concentration to ensure statistical reliability. The percentage of cell viability was calculated relative to the control groups. Data were expressed as mean ± SD from five independent biological replicates.

### Colony formation assa*y*

In the Colony Formation Assay, PC-3 and WPMY-1 cells were seeded onto 6-well plates at a density of 1,000 cells per well. Subsequently, the cells were exposed to ATBC at concentrations of 0, 2.5, 5, 10, 20, and 40 µM for a duration of 24 hours. The cells were incubated at 37°C and permitted to proliferate for a period of two weeks. The experiment was performed independently five times (n = 5). Following this incubation period, the resulting cell colonies were fixed using cold methanol for 30 minutes and subsequently stained with 0.1% crystal violet. Data are expressed as mean ± SD from five independent biological replicates. Data were expressed as mean ± SD from five independent biological replicates.

### Transwell assay and scratch assay

For the scratch assay, after cell adherence, a 200 μL pipette tip was used to create a scratch in a 6-well plate. The experiment was performed independently five times (n = 5). The cells were subsequently washed three times with PBS. Following this, the cells were cultured with varying concentrations of ATBC (containing 1% fetal bovine serum). An inverted microscope was employed to observe the changes in cell migration at 0 hours and 24 hours in each culture plate. For the invasion assay, a layer of Matrigel was first applied to the upper chamber. Subsequently, cells treated with different concentrations of ATBC for 24 hours were resuspended in serum-free medium. Next, 3 × 10^4^ cells were transferred into the upper chamber, and 500 μL of complete medium was added to the lower chamber. After 24 hours, the cells in the upper chamber were removed, fixed with 4% paraformaldehyde for 30 minutes, and then stained with crystal violet for 20 minutes. An inverted microscope was used for observation and image acquisition. Data were expressed as mean ± SD from five independent biological replicates.

### qRT-PCR analysis of hub genes mRNA expression

Total RNA was extracted from WPMY-1 cells with varying ATBC concentrations (0, 2.5, 5, and 10 μM) using the HiPure Total RNA Micro Kit. The experiment was performed independently five times (n = 5), with each biological replicate yielding one RNA extraction. Following the manufacturer’s protocol, RNA isolation was conducted, followed by DNase I treatment. The concentration and purity of the total RNA were assessed at 260 nm using a Nanodrop spectrophotometer. For the reverse transcription (RT) reaction, complementary DNA (cDNA) was synthesized from 1 mg of total RNA utilizing the HIFI cDNA synthesis kit. qRT-PCR amplification was carried out using an Applied Biosystems QuantStudio 6 Flex qRT-PCR system with the UltraSYBR Mixture. Relative mRNA levels were calculated and normalized to GAPDH expression. Data were expressed as mean ± SD from five independent biological replicates. The sequences of the forward and reverse primers employed are detailed in **Supplementary S1**.

### Molecular docking

The structures of ATBC were retrieved from the PubChem website. The EZH2, PRMT5, and DNMT1 were retrieved from the PDB database (https://www.rcsb.org/), and PyMol software was used to remove water and ligand structures. AutoDock Vina was utilized to dock EZH2, PRMT5, and DNMT1 with ATBC, resulting in the acquisition of an affinity value. Expressed in kilocalories per mole, this affinity value quantifies the binding strength between two molecules, where lower energy signifies a more stable ligand-receptor interaction. In general, a docking energy value below −5.0 kcal/mol indicate stable binding. Subsequently, PyMol was utilized to visualize the molecular docking interactions.

### Statistical analysis

All values are reported as the mean ± standard deviation (SD). Statistical analyses and significance testing were performed using R software and GraphPad. Statistical differences between two groups were assessed using either the Independent-sample t-test. For multiple concentration comparisons, one-way analysis of variance (ANOVA) followed by Dunnett’s *post hoc* test (all ATBC concentrations compared with the control group) was used to correct for multiple comparisons. Normality was assessed using the Shapiro–Wilk test, and nonparametric methods (Wilcoxon rank-sum test) were employed when the normality assumption was not met. A P-value below 0.05 was deemed statistically significant.

## Results

### Identification of targets of ATBC and PCa

In the present study, we utilized the extensive datasets available in three GEO databases to identify 4,187 differentially expressed genes (DEGs) that demonstrated a significant correlation with PCa (**Figure 1**). Following this, we conducted a thorough screening process, initially identifying 275 targets associated with ATBC from the STITCH, Swiss Target Prediction, and ChEMBL databases (**Figures 2A, B**). Through careful integration and the removal of redundant entries among these genes, we found 45 intersecting candidates were regarded as potential targets for mediating the effects of ATBC on PCa. The Venn diagram presented demonstrates the relationship between the potential genes of ATBC and PCa (**Figures 2C, D**).

**Figure 1:**
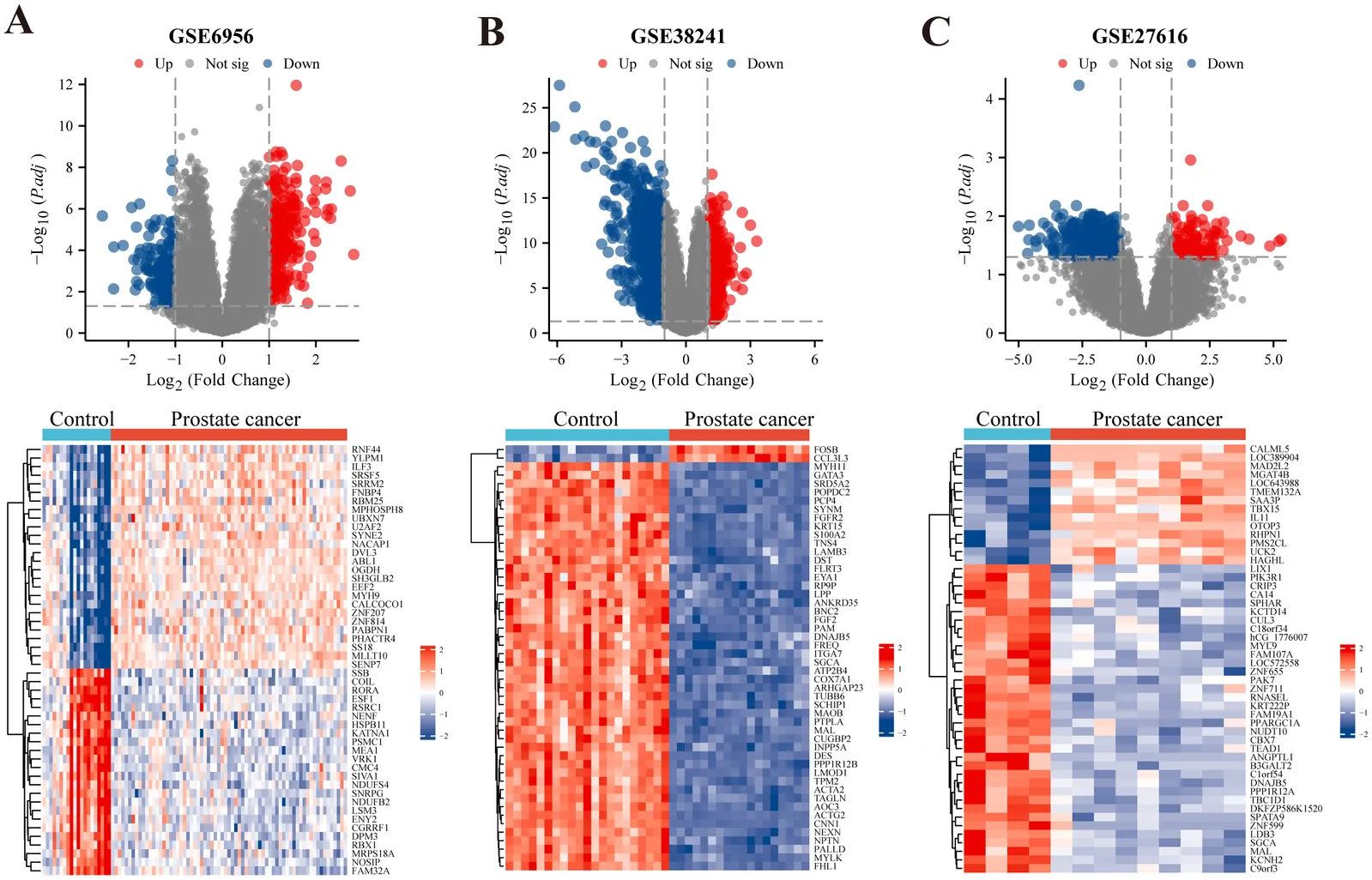
Heatmap and volcano plot of the DEGs in PCa. **(A)** GSE6956; **(B)** GSE38241; **(C)** GSE27616.

**Figure 2:**
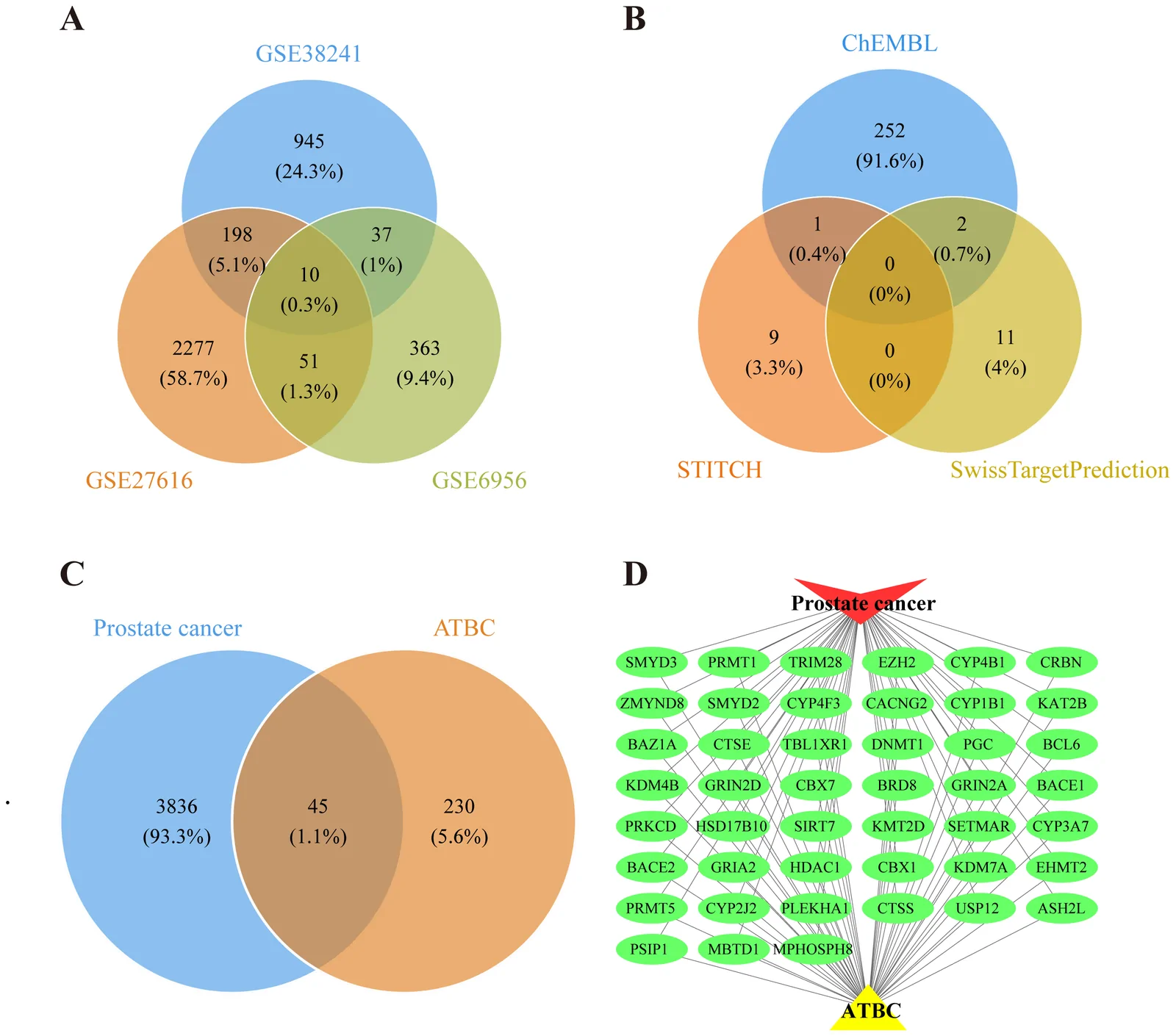
Venn diagram of the targets of ATBC and PCa. **(A)** The Venn diagram of DEGs from GEO; **(B)** The Venn diagram of targets associated with ATBC from STITCH, Swiss Target Prediction, and ChEMBL databases; **(C)** The Venn diagram of intersecting targets between ATBC and PCa; **(D)** Disease-Targets-Toxic components network diagram.

### Go and KEGG enrichment analysis

We performed a comprehensive GO analysis on 45 human targets, identifying 367 significant GO terms: 178 biological processes (BP), 70 cellular components (CC), and 119 molecular functions (MF). We ranked these terms by P value and highlighted the top 10 from each category in an enrichment analysis diagram. Additionally, KEGG analysis revealed 20 enriched signaling pathways, summarized in a bubble chart and histogram, highlighting the top 10 pathways by significance. The GO and KEGG analyses findings suggested that the BP predominantly involve histone modification, methylation, macromolecule methylation, peptidyl-lysine modification, regulation of DNA metabolic process, protein alkylation, protein methylation, histone methylation peptidyl-lysine methylation, and histone lysine methylation (**Figure 3A**). The CC identified include methyltransferase complex, heterochromatin, postsynaptic specialization membrane, postsynaptic density membrane, histone methyltransferase complex, neurotransmitter receptor complex, ionotropic glutamate receptor complex, integral component of postsynaptic density membrane, hippocampal mossy fiber to CA3 synapse, and pericentric heterochromatin (**Figure 3B**). The MF are likely associated with transcription coregulator activity, transferase activity, transferring one-carbon groups, methyltransferase activity, S-adenosylmethionine-dependent methyltransferase activity, N-methyltransferase activity, protein methyltransferase activity, histone methyltransferase activity, lysine N-methyltransferase activity, protein-lysine N-methyltransferase activity, and histone-lysine N-methyltransferase activity (**Figure 3C**). The KEGG signaling pathways were notably concentrated in various pathways, including the Notch signaling pathway, Proteoglycans in cancer, cGMP-PKG signaling pathway, ECM-receptor interaction, Signaling pathways regulating pluripotency of stem cells, Thyroid hormone signaling pathway, Cardiac muscle contraction, Complement and coagulation cascades, Prostate cancer, Metabolism of xenobiotics by cytochrome P450, Drug metabolism- cytochrome P450 (**Figure 3D**).

**Figure 3:**
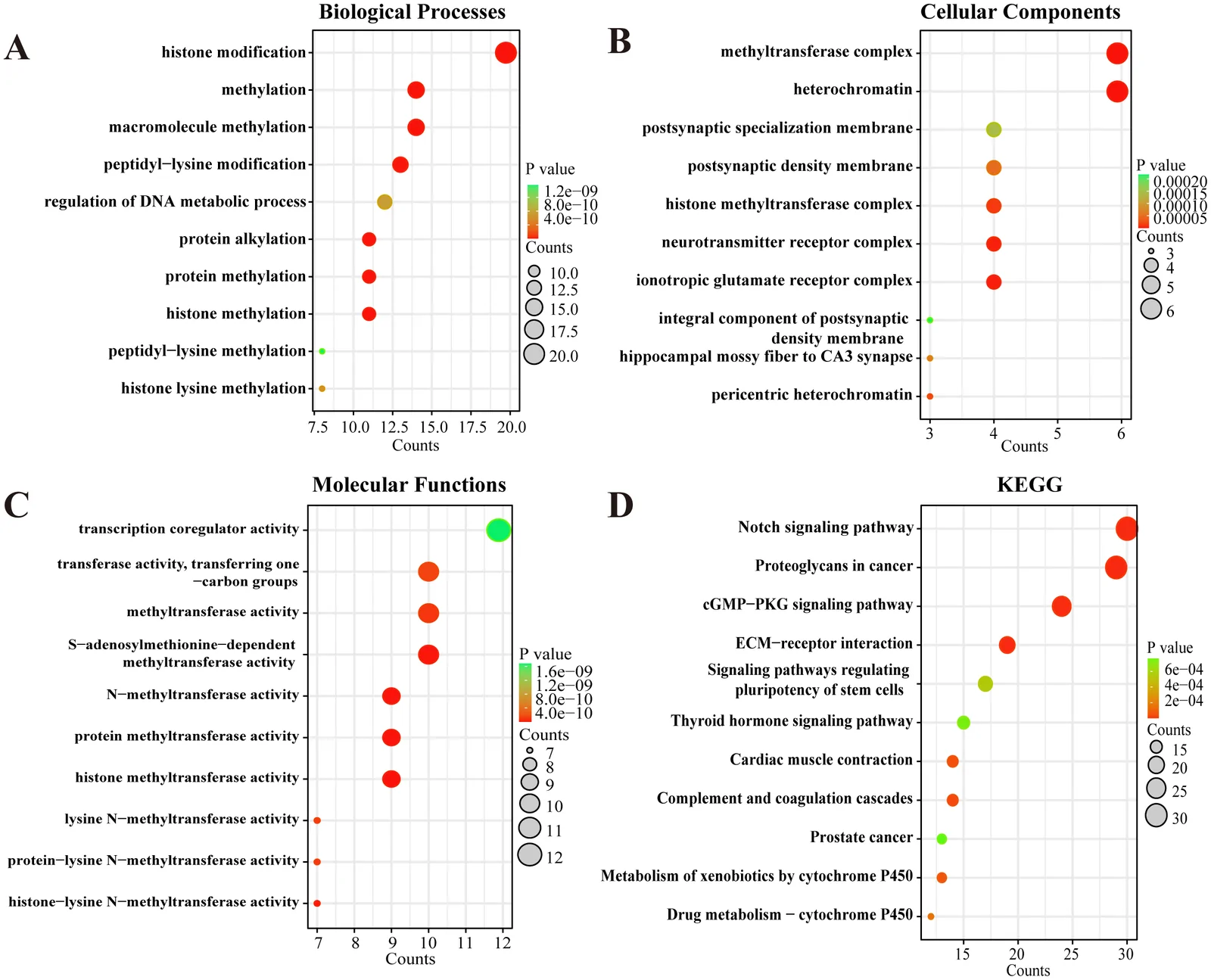
GO and KEGG enrichment analysis. **(A)** biological processes; **(B)** cellular components. **(C)** molecular functions; **(D)** The KEGG of pathway enrichment analysis.

### Interaction network of potential targets

Using the STRING database, we constructed a PPI network with 45 nodes and 125 edges (**Supplementary 2**). We then used Cytoscape to analyze the network’s topology and create an optimized diagram. This visualization illustrates complex interactions and highlights node importance through size and color, with larger and darker nodes indicating higher degree values. This visualization clearly depicts the complex interdependencies within the group, highlighting EZH2, HDAC1, EHMT2, and DNMT1 as key targets with the highest degree value, emphasized by their larger size and intense red color, indicating their crucial role in the PPI network (**Figure 4A**). We identified 10 key ATBC targets affecting PCa and used Cytoscape to create a focused network diagram showcasing their interactions (**Figure 4B**).

**Figure 4:**
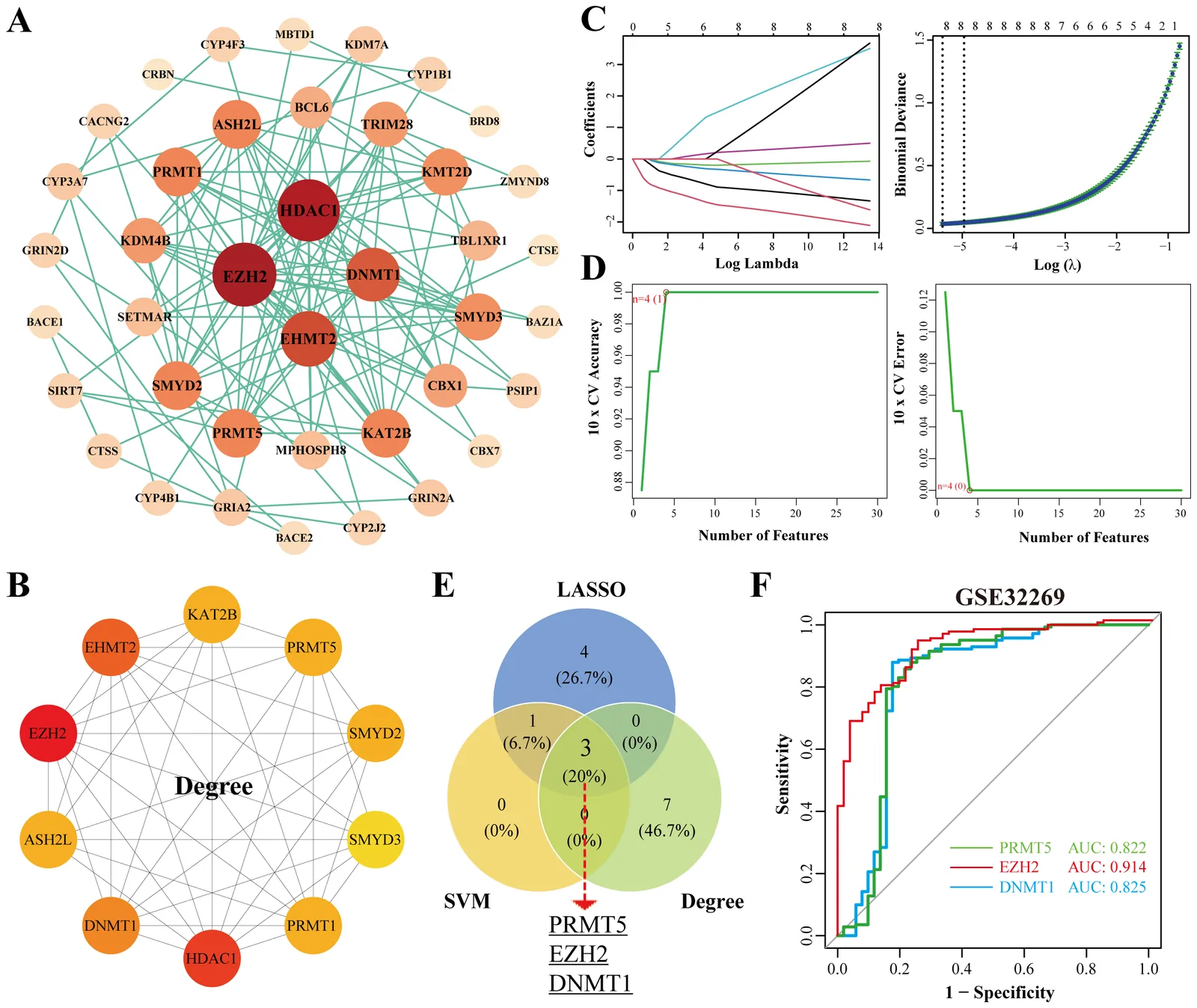
Screening for characteristic genes by machine learning strategies. **(A)** PPI network of the intersecting targets; **(B)** The top 10 genes of Degree algorithm; **(C)** The 8 characteristic genes of the LASSO algorithm; **(D)** The 4 key genes of the SVM-RFE algorithm; **(E)** Venn diagram displaying 3 overlapping genes obtained by LASSO, SVM-RFE, and Degree algorithms; **(F)** ROC and AUC for characteristic genes in GEO datasets. AUC = area under the curve, ROC = receiver operating characteristic.

### Machine learning predicts characteristic genes

To identify core genes of the intersecting targets, we employed two distinct machine learning algorithms to acquire differential genes. Initially, LASSO regression analysis was utilized to filter the characteristic genes, resulting in the identification of 8 overlapping feature genes (BACE1, CBX7, CYP3A7, CYP4B1, PRMT5, EZH2, MBTD1, and DNMT1) were identified (**Figure 4C**). Subsequently, the SVM-RFE algorithm was applied to determine the disease-specific characteristic genes, illustrating the change curve of the cross-validation error for each gene. Finally, we selected the top 4 genes (EZH2, CBX7, PRMT5, and DNMT1) (**Figure 4D**). Ultimately, we identified the intersection of two algorithms and the Degree algorithm in PPI network, finally obtained 3 characteristic genes (EZH2, DNMT1, and PRMT5) (**Figure 4E**). To validate these findings, an independent validation analysis was performed using the GSE32269 dataset. Results showed that the three characteristic genes consistently exhibited significant diagnostic efficacy, the ROC analysis confirmed the diagnostic value of the three characteristic genes: PRMT5 (AUC = 0.822), DNMT1 (AUC = 0.914), and EZH2 (AUC = 0.825) (**Figure 4F**).

### TCGA-PRAD and HPA validation

The TCGA-PRAD was employed to validate the expression of 3 hub genes, the findings indicated that a clear correlation between hub genes (**Figure 5A**). Meanwhile, EZH2, DNMT1, and PRMT5 were significantly overexpressed in patients with TCGA-PRAD tumors (**Figure 5B**). Furthermore, ROC analysis demonstrated a strong predictive capability for EZH2, DNMT1, and PRMT5 within the TCGA-PRAD dataset, with AUC values exceeding 0.75 (**Figure 5C**). In addition, immunohistochemical data for normal prostate tissue and PCa tissue, stained with antibodies specific to EZH2, DNMT1, and PRMT5 (CAB012459, CAB002694, and CAB009589, respectively), were sourced from the HPA database. The immunohistochemical staining results revealed that PCa tissue exhibited more intense staining in comparison to normal tissue, and suggesting a significant overexpression of these three proteins in PCa tissue (**Figure 5D**).

**Figure 5:**
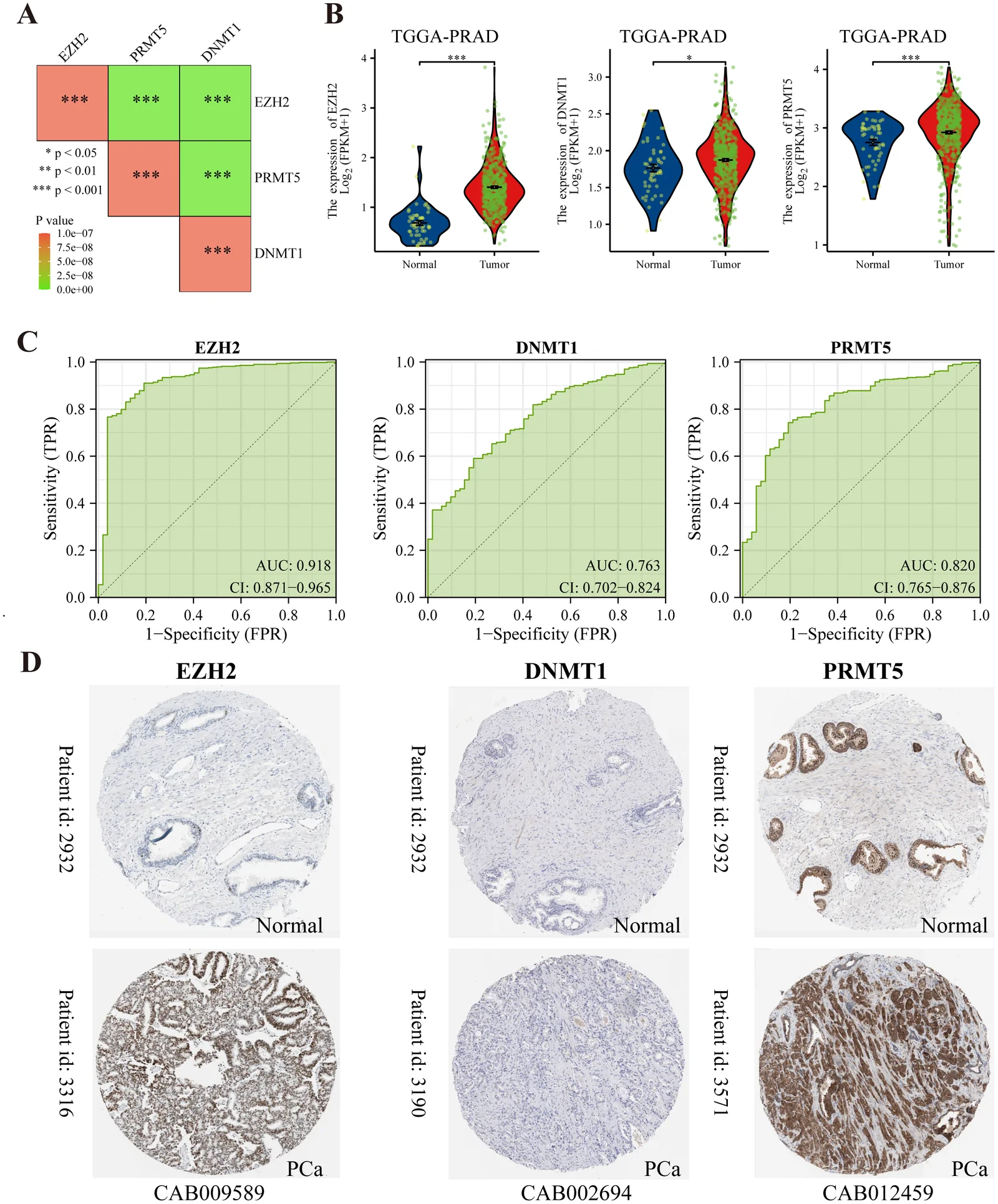
Expression and validation of hub genes in TCGA-PRAD and HPA. **(A)** Correlation diagram of EZH2, DNMT1, and PRMT5; **(B)** The expression patterns of EZH2, DNMT1, and PRMT5 in the TCGA-PRAD cohort; **(C)** The AUC values of EZH2, DNMT1, and PRMT5; **(D)** Immunohistochemical staining of ZH2, DNMT1, and PRMT5 in normal and PCa tissue.

### Immune infiltration of ATBC in PCa

We employed CIBERSORT to evaluate the immune infiltration and observed a significant difference in the PCa immune microenvironment (**Figures 6A, C**). To explore whether the hub genes associated with ATBC influence the immune microenvironment, we analyzed immune cell infiltration based on the expression levels of EZH2, DNMT1, and PRMT5 in PCa samples. Next, we conducted a correlation analysis between immune cells and hub genes, with red indicating positive correlation, blue indicating negative, and darker shades representing stronger correlations. The results show PRMT5 was positively correlated with Dendritic cells resting, Macrophages M2, Mast cells resting, NK cells activated, T cells CD4 memory resting, T cells follicular helper, T cells gamma delta, and negatively correlated with Macrophages M0, NK cells resting, T cells CD4 memory activated, T cells CD4 naive, T cells regulatory (Tregs); EZH2 was positively correlated with Macrophages M2, and negatively correlated with NK cells resting; DNMT1 was positively correlated with Dendritic cells activated, Dendritic cells resting, Macrophages M2, Mast cells resting, NK cells activated, T cells CD4 memory resting, T cells follicular helper, T cells gamma delta, and negatively correlated with Macrophages M0, NK cells resting, T cells CD4 memory activated, T cells CD4 naive, T cells regulatory (Tregs) (**Figure 6D**). These immune cells are crucial components of the immune response and their increased infiltration implies that these hub genes may alter the immune landscape in PCa. Our findings suggest that ATBC-related hub genes may influence immune responses within PCa.

**Figure 6:**
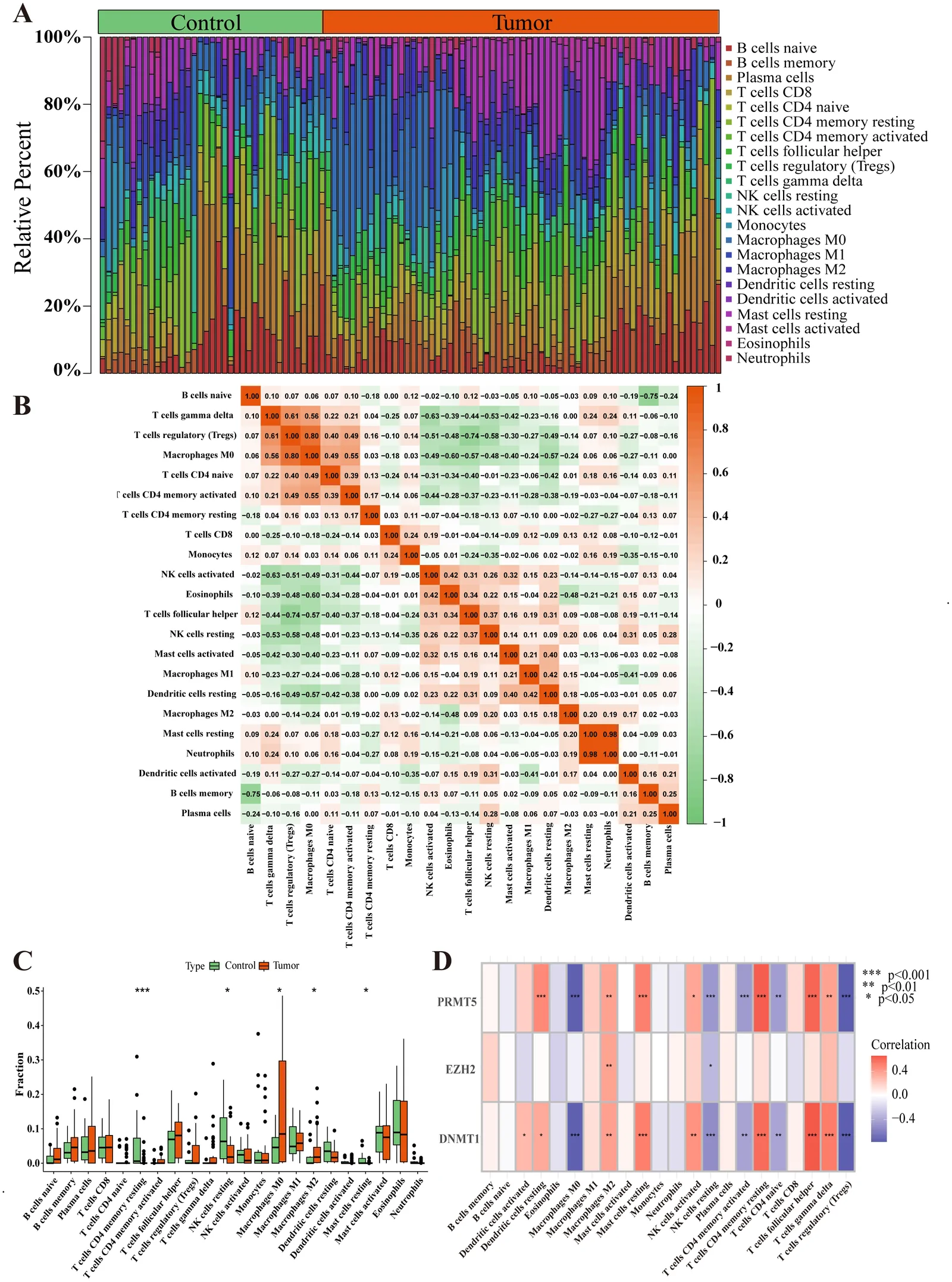
Visualization and evaluation of immune cell infiltration. **(A)** The proportion of different immune cell infiltration in PCa samples; **(B)** Correlation analysis of 22 kinds of immune cells in PCa samples; **(C)** Box plots of 22 immune cell types infiltration between normal and PCa samples; **(D)** Immune cells correlation scatter plot of EZH2, DNMT1, and PRMT5 genes.

### Single-cell analysis reveals hub gene expression patterns within tumor microenvironment

Single-cell RNA sequencing of the GSE143791 dataset identified specific cell-type expression of ATBC-related genes in PCa. UMAP clustering revealed distinct subpopulations within PCa tissues, which were annotated into precise cell types such as B cells, CD4T cells, CD8T cells, DC cells, Endothelial cells, Erythroid cells, Malignant cells, Monor/Macro cells, NK cells, Osteoblasts cells, Osteoclasts cells, Pericytes cells, Plasma cells, Progenitor cells, pDC cells (**Figures 7A, B**). Marker genes confirmed the special expression profiles and biological relevance of these subpopulations (**Figure 7C**). The varying proportions of cellular subgroups underscore the PCa microenvironment’s complexity and heterogeneity (**Figure 7D**). UMAP plots were used to visualize the expression levels and distribution of EZH2, DNMT1, and PRMT5. EZH2 was notably upregulated in Erythroid and Progenitor cells, DNMT1 in CD4T, CD8T, Monor/Macro, Erythroid, and Progenitor cells, and PRMT5 in Erythroid and Progenitor cells. These findings suggest that ATBC may affect cancer development by modulating cell populations within PCa microenvironment (**Figure 7E**).

**Figure 7:**
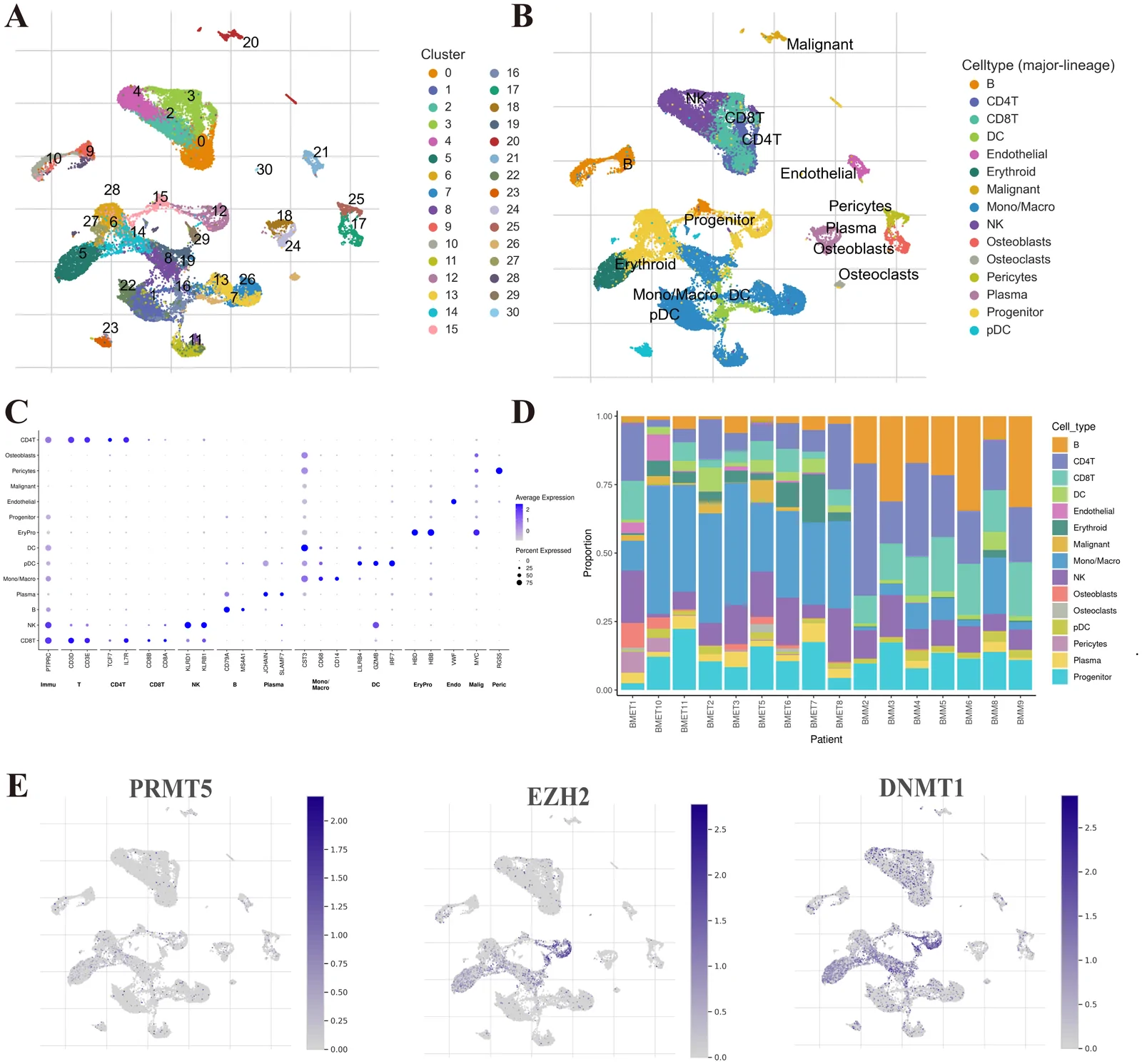
Single-cell RNA sequencing analysis from PCa dataset (GSE143791). **(A)** UMAP visualization delineating cell clusters within PCa samples, with different colors represent distinct clusters; **(B)** Detailed annotation of clusters; **(C)** Heatmap depicting marker genes specific to different cell cluster; **(D)** Bar plot illustrating the distribution of cell cluster within the PCa tumor microenvironment; **(E)** UMAP plots depicting the expression pattern of key gene from single-cell data.

### Identification and functional analysis of hub genes KO-responsive genes

To further elucidate the roles of these core genes, we performed functional enrichment analysis on differentially expressed genes following the virtual knock out of DNMT1, EZH2, and PRMT5. The results indicated that these three genes are primarily involved in maintaining cellular component assembly and the organizational structure of the extracellular matrix (ECM). Specifically, they are critical for the structural integrity of collagen-containing extracellular matrices and for cell–matrix adhesion (**Figure 8**). These findings suggest that DNMT1, EZH2, and PRMT5 may play important roles in maintaining the structural and epigenetic homeostasis of normal prostate stromal cells. Since the virtual knockout experiments were conducted on prostate cancer single-cell datasets without ATBC induction, this study only hypothesizes a potential correlation between these regulatory genes and stromal homeostasis. We speculate that ATBC may exert its toxic effects by disrupting the homeostatic functions of these regulatory genes, thereby leading to structural and functional disruption of stromal cells, a mechanism consistent with the cytotoxic effects observed in our *in vitro* experiments.

**Figure 8:**
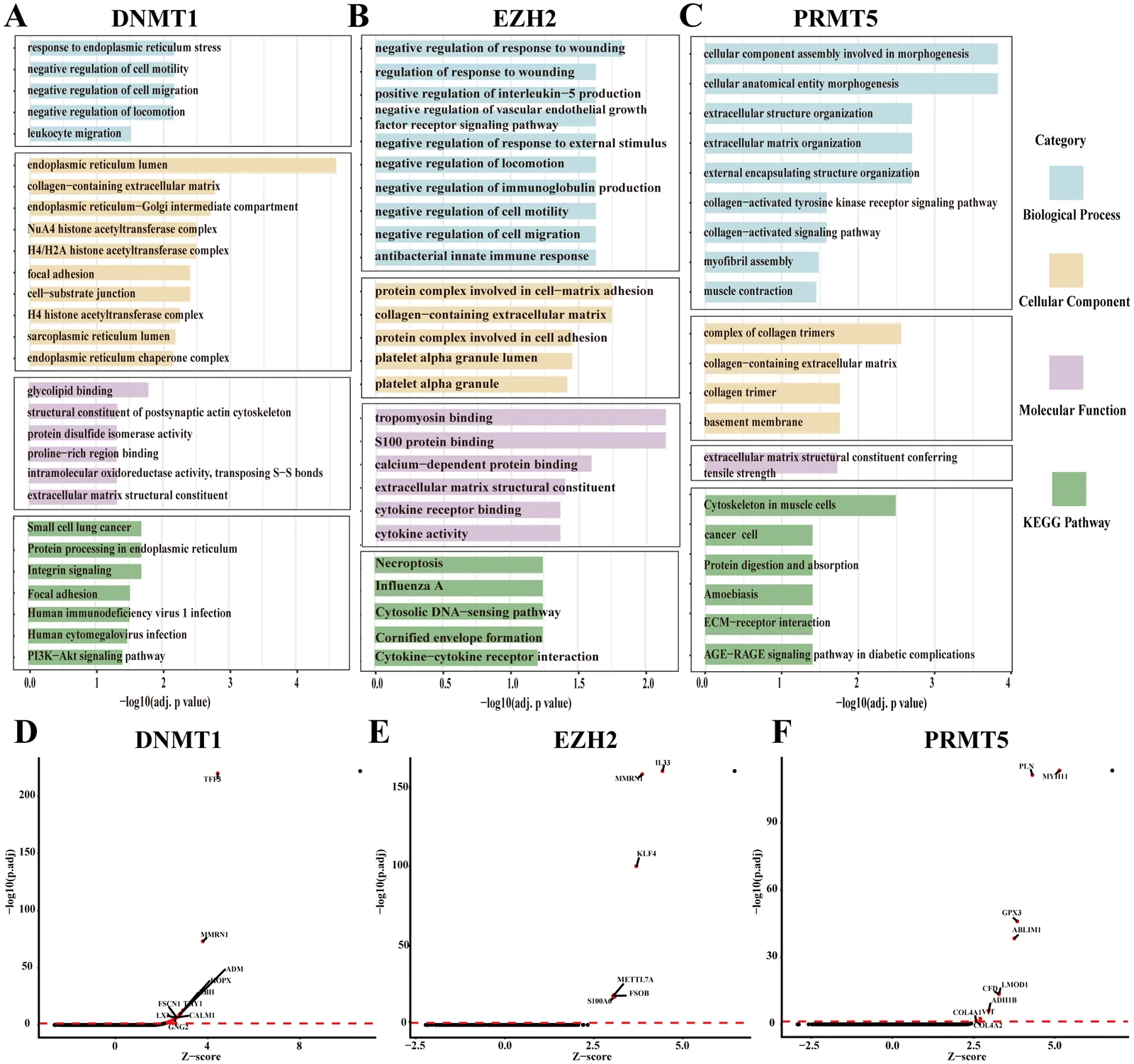
Analysis of potential functional changes following virtual knock out of core genes. **(A–C)** GO and KEGG enrichment analyses of the top 20 significantly altered genes; **(D–F)** Volcano plots showing significantly dysregulated genes after virtual knock out of core genes.

### Toxic effects of ATBC on prostate cells

To examine the effects of ATBC on prostate cells, we utilized cell experiments comprising WPMY-1 cell lines. The cells were exposed to ATBC at concentrations of 0, 2.5, 5, 10, 20, and 40 μM, and their proliferation was evaluated using CCK-8 and colony formation assays. The proliferation capacity of WPMY-1 cells decreased linearly even at low ATBC concentrations (**Figure 9A**). Notably, ATBC demonstrated significant inhibitory effects at a concentration of 5 μM, implying that low-dose ATBC may affect normal prostate tissue homeostasis. The colony formation assay further revealed that low concentrations of ATBC significantly inhibited the colony formation of WPMY-1 cells (**Figures 9B, C**). The results of the wound healing assay demonstrated that ATBC inhibited the migration of WPMY-1 cells in a concentration-dependent manner. Furthermore, the number of invasive cells was significantly reduced, indicating that ATBC suppresses the migration and invasion of WPMY-1 cells (**Figures 9D–G**). To investigate the potential targets underlying the effects of ATBC, we performed a comprehensive analysis of gene expression using qRT-PCR, focusing specifically on EZH2, PRMT5, and DNMT1, across ATBC concentrations ranging from 0 μM to 10 μM. These results showed that ATBC altered the expression of these three key genes (*p* < 0.05), which may affect the progression of PCa (**Figures 9H–J**).

**Figure 9:**
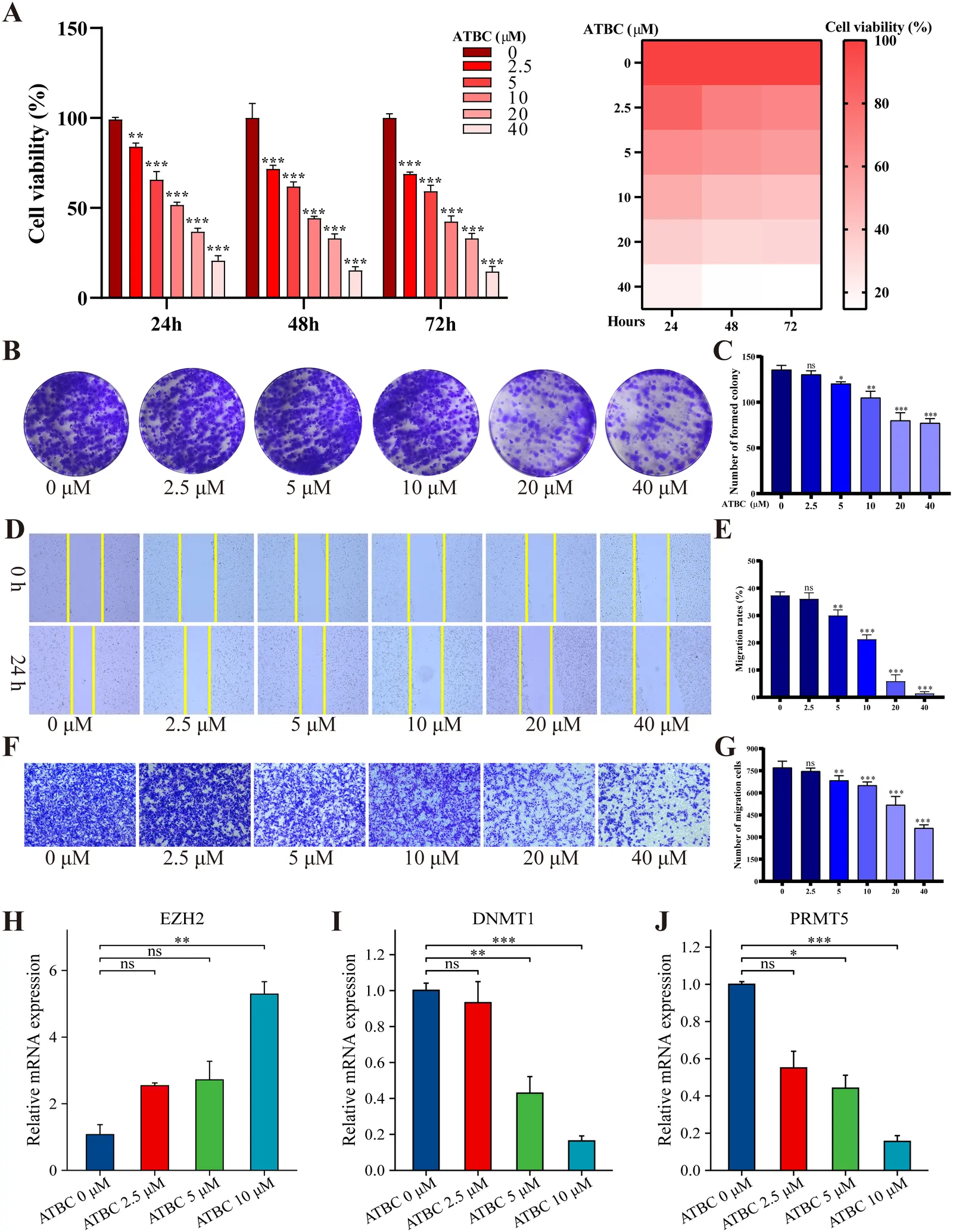
The effects of ATBC on WPMY-1 cells. **(A)** Cell viability of WPMY-1 cells. n=5. **(B, C)** Colony forming ability of WPMY-1 cells. n=5. **(D, E)** Cell migration determined by wound healing assay. Scale bar = 20 μm. n=5. **(F, G)** Cell invasion determined by Transwell assay. Scale bar = 20 μm. n=5. **(H–J)** Relative expression of the EZH2, PRMT5, and DNMT1. n=5. Data are shown as the mean ± SEM or SD. ns: *P >*0.05, *p < 0.05, **p < 0.01, ***p < 0.001.

### ATBC promotes the proliferation and migration of PCa cells *in vitro*

To evaluate the potential of ATBC to promote prostate cancer progression, we further investigated its effects on prostate cancer cells. CCK-8 and colony formation assays demonstrated that ATBC promoted the proliferation of PC-3 cells starting at a concentration of 5 μM (**Figures 10A–C**). Transwell and wound-healing assays indicated that ATBC significantly enhanced the migration and invasion capabilities of prostate cancer cells (**Figures 10D–G**).

**Figure 10:**
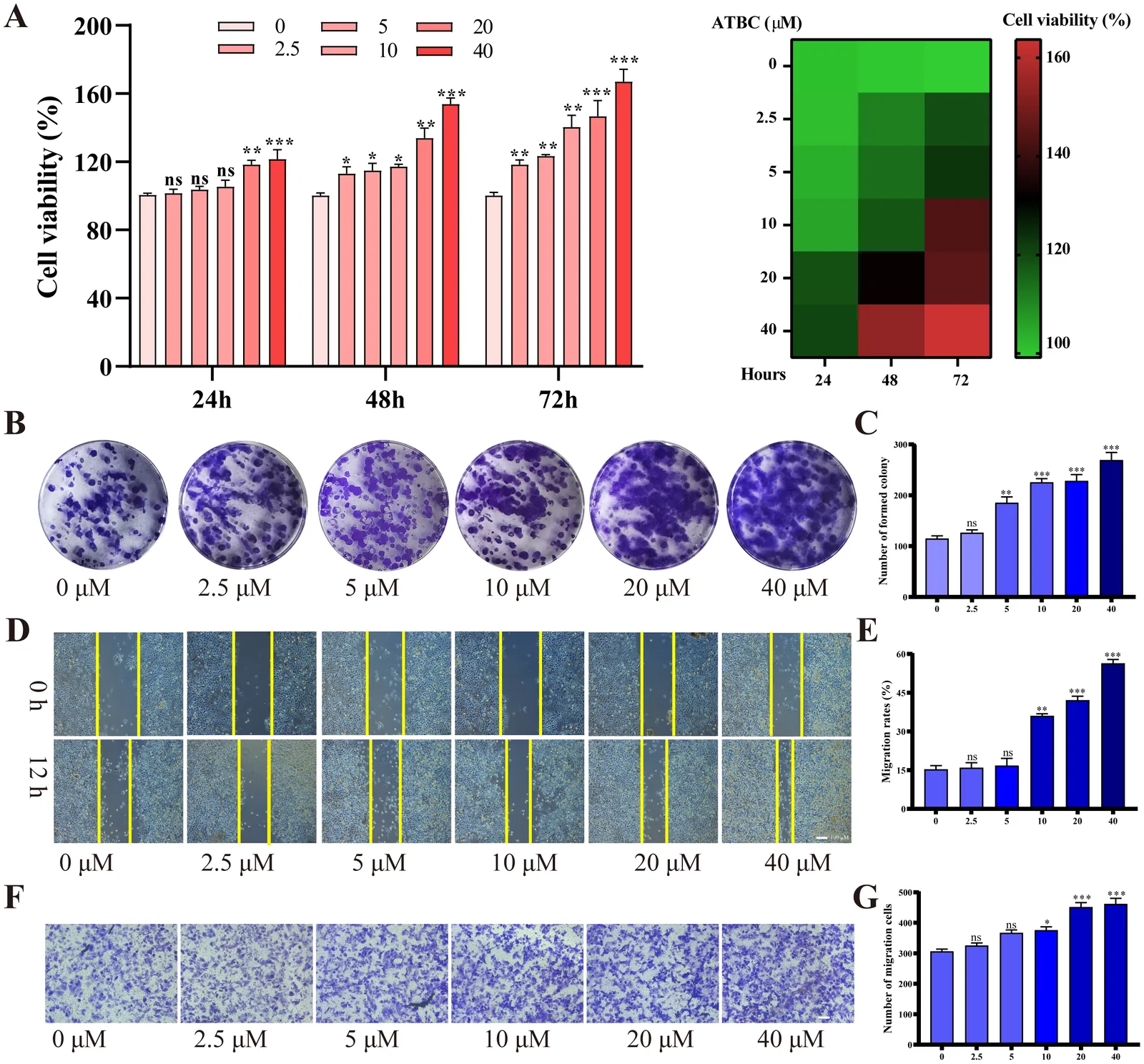
The effects of ATBC on PC-3 cells. **(A)** Cell viability of PC-3 cells. n=5. **(B, C)** Colony forming ability of PC-3 cells. **(D, E)** Cell migration determined by wound healing assay. Scale bar = 20 μm. n=5. **(F, G)** Cell invasion determined by Transwell assay. Scale bar = 20 μm. n=5. Data are shown as the mean ± SEM or SD. ns: *P >*0.05, *p < 0.05, **p < 0.01,***p < 0.001.

### Molecular docking situation

To further substantiate the binding activity and identify the binding sites of ATBC with specific genes, molecular docking analysis was performed to investigate the interactions between ATBC and three key targets: EZH2, PRMT5, and DNMT1. Our docking simulations revealed low binding energies, falling below -5 kcal/mol, the result highlights the strong affinity and significant role in the molecular mechanisms influencing PCa. To elucidate the intricate binding configurations in greater detail, the visualization of PyMOL illustrated the lowest energy binding conformation. Visualization of the binding interfaces between ATBC and the target proteins demonstrated stable binding sites within these models. These findings propose that EZH2 (-7.2 kcal/mol), DNMT1 (-5.7 kcal/mol) and PRMT5 (-6.5 kcal/mol) identified as ATBC prospective targets (**Figure 11**).

**Figure 11:**
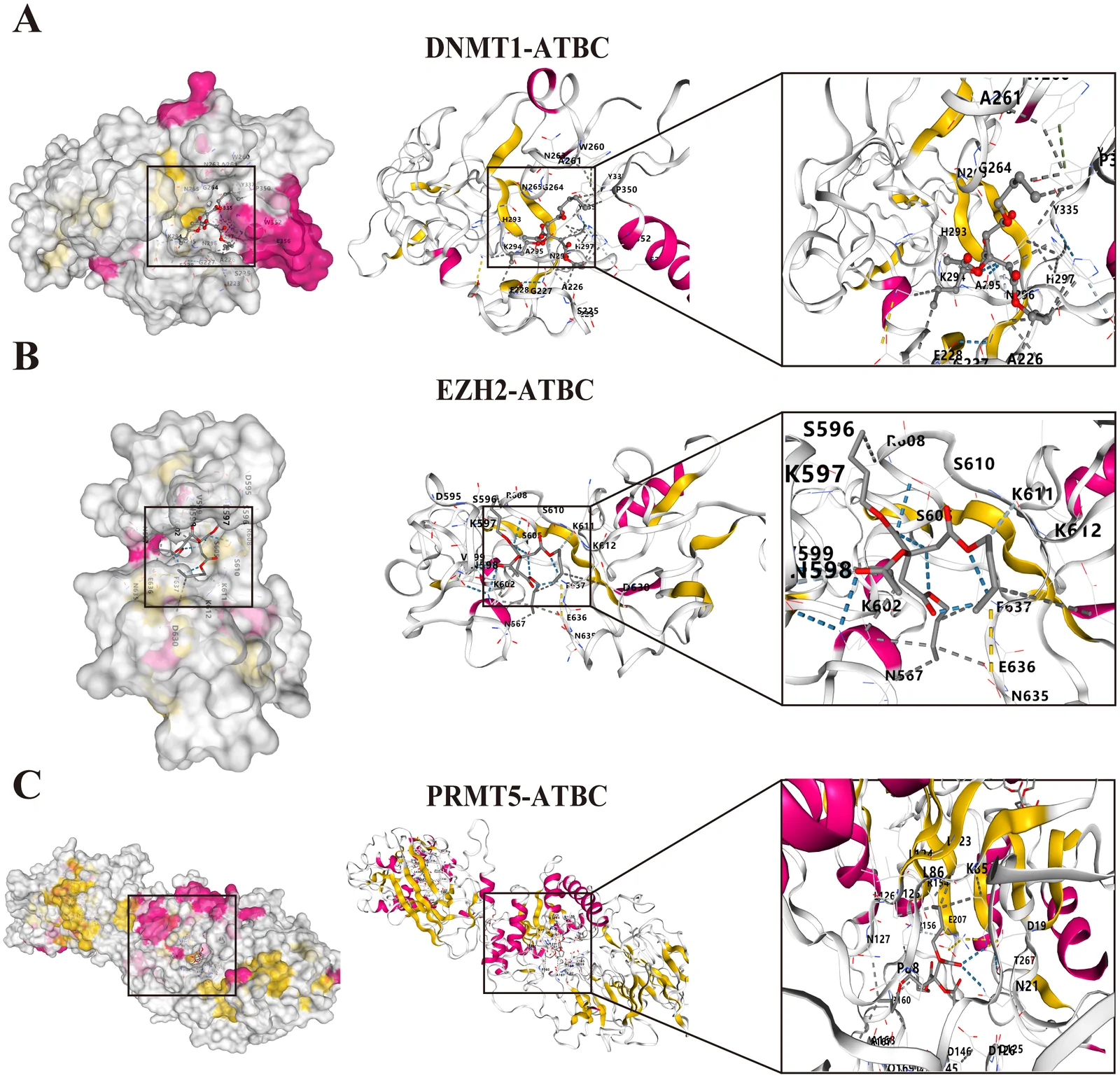
Illustrates the molecular docking pattern. **(A)** DNMT1-ATBC; **(B)** EZH2-ATBC; **(C)** PRMT5-ATBC.

## Discussion

In this study, we conducted a meticulous screening of 45 potential targets associated with the impact of ATBC on PCa by utilize PubChem, GEO, ChEMBL, STITCH, and Swiss Target Prediction databases. Through the application of the STRING and Cytoscape, we developed an interaction network of these candidate genes. Subsequently, combining machine learning with PCR validation, we identified and confirmed three key targets: EZH2, PRMT5, and DNMT1. These targets reflect ATBC’s impact on PCa and may offer novel insights into the potential biological mechanisms.

Enhancer of Zeste Homolog 2 (EZH2) plays a critical role in gene expression regulation through its activity as a histone methyltransferase within the Polycomb Repressive Complex 2 (PRC2) (26). Its principal function involves the trimethylation of histone H3K27me3, resulting in the formation of repressive chromatin states. This epigenetic modification is essential for the silencing of tumor suppressor genes, thereby promoting oncogenesis in various cancers, including prostate cancer, breast cancer, and glioblastoma (27–29). The dysregulated expression of EZH2 has been associated with poor prognosis and enhanced metastatic potential in numerous malignancies, underscoring its role as a negative prognostic indicator (30). Additionally, EZH2 affects the expression of non-histone proteins, thereby influencing various processes such as proliferation and apoptosis (31). For example, EZH2 has been demonstrated to interact with transcription factors and modulate their activity independently of its methyltransferase function (32). The interaction between EZH2 and various signaling pathways is a key aspect of its role in cancer progression. One of the most significant interactions is with the Notch pathway, which is frequently activated in many cancer types, including Chronic lymphocytic leukemia and Glioblastoma (33). This pathway is crucial for regulating cell growth, survival, and metabolism, and its dysregulation is often linked to poor clinical outcomes (34). Moreover, the interplay between EZH2 and signaling pathways, including the thyroid hormone and PI3K/Akt pathway, underscores its multifaceted role in cancer biology (35). Overall, EZH2’s interactions with signaling pathways, notably the Notch and PI3K/Akt pathways are pivotal in cancer progression (36). Dysregulation of the Notch pathway promotes EZH2 phosphorylation, increasing its oncogenic potential. Understanding these interactions may lead to novel cancer therapies, especially in tumors with abnormal EZH2 and Notch activation.

Protein arginine methyltransferase 5 (PRMT5) is a significant member of the protein arginine methyltransferase family. Recent research has demonstrated that PRMT5 is overexpressed in various malignancies, such as prostate, breast, and colorectal cancers, suggesting its function as an oncogene (37, 38). PRMT5 exerts its biological effects through diverse mechanisms, primarily by modifying histones and non-histone proteins via arginine methylation (39). PRMT5 interacts with the androgen receptor (AR) in PCa, boosting AR transcriptional activity and driving castration-resistant progression (40). PRMT5 also regulates the expression of epithelial-mesenchymal transition (EMT)-related markers, thereby contributing to the invasive capabilities of Lung cancer (41). Studies have indicated that elevated levels of PRMT5 expression are associated with poorer survival outcomes (42). This association has been attributed to the role of PRMT5 in promoting oncogenic pathways and inhibiting the expression of tumor suppressor genes, thereby facilitating cancer progression (43). Targeting PRMT5 and EZH2 can synergistically promote progression in colorectal cancer (CRC), the possibility that combining with PRMT5 and EZH2 may represent a promising strategy for CRC management (44).

DNA methyltransferase 1 (DNMT1) is a crucial enzyme involved in maintaining DNA methylation patterns, which are essential for regulating gene expression. DNMT1 levels were significantly higher in hormone-naïve high-grade PCa compared to low-grade cases, and further elevated in hormonally treated and CRPC (45). DNMT1 levels not only was highly abundant in PC-3, CWR22, LNCaP and PC-3 cells, also correlate with increased methylation of tumor suppressor genes, leading to their silencing and facilitating cancer cell proliferation and survival (46). Additionally, the interaction between DNMT1 and other epigenetic regulators, including EZH2, has been implicated in the enhancement of PCa migration and invasion, underscoring the enzyme’s multifaceted role in tumorigenesis (47). Additionally, DNMT1’s interaction with miRNAs has been identified as a regulatory mechanism in gene silencing, such as DNMT-1 enhances miR-27-3p expression to inhibit the Notch pathway activation in LSCC (48).

Tumor microenvironment (TME) consists of various cellular and extracellular components that collectively influence cancer progression, metastasis, and therapeutic responses. The interaction immune cells of TME become increasingly complex in PCa progresses (49). This complexity underscores the necessity of understanding the mechanisms that govern these interactions, particularly the roles of key targets related to ATBC such as EZH2, DNMT1, and PRMT5 in PCa, which are most closely related with NK cells, Th2 cells, Tcm cells, and pDC cells. By elucidating the interactions between the ATBC, immune cells and these critical targets, we can identify potential immune cells for therapeutic intervention.

Applying network toxicology to microplastics can speed up identifying crucial molecular targets, leading to better regulatory standards and preventive measures. We identified key genes and pathways involved in ATBC-induced cytotoxicity and pro-tumorigenic effects, ATBC seems to influence the onset and progression of PCa by affecting oncogenic genes and immune function. Nevertheless, some limitations still persist. Firstly, the present toxicity study was restricted to the experiments performed in WPMY-1 and PC-3 cells. Afterward, a definitive correlationship between ATBC and PCa was not well demonstrated in animal models. Moreover, the effects of short-term exposure studies may not accurately simulate the long-term influence on human health. Finally, the lack of single-cell data from ATBC-exposed samples in the virtual knockout approach limits our ability to directly infer causal relationships, and future validation using ATBC-exposed single-cell RNA sequencing or *in vivo* gene knockout experiments is warranted. To further clarify the molecular mechanisms behind the effects of ATBC on PCa, it is essential to validate these targets through large-scale epidemiological studies and animal models simulating chronic, low-dose ATBC exposure.

## Data Availability

The original contributions presented in the study are included in the article/**Supplementary Material**. Further inquiries can be directed to the corresponding authors.
