# Supplementary material for: Analyzing the cytotoxicity and pro‐tumorigenic role of acetyl tributyl citrate in prostate through network toxicology and the immune microenvironment
Source: Front Immunol. 2026 Sep 8;17:1758050. doi: 10.3389/fimmu.2026.1758050 (PMC13617745; doi:10.3389/fimmu.2026.1758050)
Supplement: Supplementary file 2 [file Table1.docx]

**Table 1.** Primer sequences used for RT-qPCR

| **Genes** | **Forward (5'-3')** | **Reverse (5'-3')** |
| --- | --- | --- |
| EZH2 | GATGAAGCTGACAGAAGAGGGAA | GCATAGCAGTTTGGATTTACCGA |
| PRMT5 | CACCAGAGGACCTGAGAGATGATAT | GGGAGAATGGCTGCTTTGAT |
| DNMT1 | AAGAGCGGAGGTGTCCCAATA | CCATCAGTCTTGACGGCTTCT |
| GAPDH | GGAAGCTTGTCATCAATGGAAATC | TGATGACCCTTTTGGCTCCC |

**Table 2.** Basic information on the datasets from GEO database

| Accession | Samples | Tissue information | Platforms | DEGs |  |
| --- | --- | --- | --- | --- | --- |
| GSE6956 | 89 | 20 prostate normal tissues, 69 prostate cancer tissues | GPL571 | 461 | Homo sapiens |
| GSE38241 | 39 | 18 prostate normal tissues, 21 prostate cancer tissues | GPL4133 | 1190 | Homo sapiens |
| GSE27616 | 13 | 4 prostate normal tissues, 7 prostate cancer tissues | GPL4133 | 2536 | Homo sapiens |
