# Supplementary material for: Analyzing the cytotoxicity and pro‐tumorigenic role of acetyl tributyl citrate in prostate through network toxicology and the immune microenvironment
Source: Front Immunol. 2026 Sep 8;17:1758050. doi: 10.3389/fimmu.2026.1758050 (PMC13617745; doi:10.3389/fimmu.2026.1758050)
Supplement: Supplementary file 3 [file DataSheet1.docx]

**Figure 1.** Cluster analysis of the PPI network.
